# Cardiac late events in German breast cancer patients: a validation study on the agreement between patient self-reports and information from physicians

**DOI:** 10.1186/s12872-018-0961-7

**Published:** 2018-11-29

**Authors:** Hiltrud Merzenich, Maria Blettner, Dorothea Niehoff, Lukas Schwentner, Marcus Schmidt, Margit Schmitt, Daniel Wollschläger

**Affiliations:** 1 Institute of Medical Biostatistics, Epidemiology and Informatics, University Medical Center Mainz, Obere Zahlbacher Str. 69, 55131 Mainz, Germany; 2 Department of Gynecology and Obstetrics, University Hospital Ulm, Prittwitzstr. 43, 89075 Ulm, Germany; 3 Department of Obstetrics and Gynecology, University Medical Center Mainz, 55101 Mainz, Germany

**Keywords:** Breast cancer therapy, Self-reported cardiac events, Validation, Physicians’ information

## Abstract

**Background:**

Self-administered health-status questionnaires are important tools in epidemiology. The objective of the presented validation study is to measure the agreement between breast cancer patients’ self-reports and their physicians’ information on late cardiac events, and to investigate determinants of agreement. To estimate possible misclassification is an important requirement for observational studies on cardiovascular endpoints.

**Methods:**

A retrospective, multi-center cohort study included 11,982 women diagnosed with breast cancer in Germany in 1998–2008. In 2014, a questionnaire survey assessed cardiovascular risk factors and incident cardiac events after therapy. A validation study was conducted, based on a sample of 3091 breast cancer patients from two university hospitals. Among them, 2261 women (73%) sent back the questionnaire on cardiovascular events, and 1316 women gave consent to request medical records from their general practitioners. A total of 1212/1316 (92.1%) medical records could be obtained for validation. Cohen’s kappa coefficient was calculated, and multivariate regression was applied to study the influence of patient characteristics on agreement between both data sources.

**Results:**

Overall agreement for the composite endpoint of any cardiac event was 84.5% (kappa 0.35). Of 1055 breast cancer patients reporting no cardiac event, 950 (90%) had no such diagnosis in physicians’ medical records. A total of 157 breast cancer survivors indicated a cardiac event, and the same diagnosis was confirmed by GPs for 74 (47%) women. For specific diagnoses, moderate to substantial agreement of self-reports was found for myocardial infarction (kappa 0.54) and stroke (kappa 0.61). Poor to fair agreement was present for angina pectoris, valvular heart disease, arrhythmia, and congestive heart failure. Younger age, higher education and a more recent cancer diagnosis were found to be associated with greater total agreement.

**Conclusions:**

For the composite endpoint, survivors of breast cancer report the absence of cardiac disease accurately. However, for specific diagnoses, self-reported morbidity data from breast cancer patients may not fully agree with information from physicians. The agreement is moderate for acute events like myocardial infarction and stroke, but poor to fair for chronic diseases.

**Electronic supplementary material:**

The online version of this article (10.1186/s12872-018-0961-7) contains supplementary material, which is available to authorized users.

## Background

In epidemiological studies, information about chronic diseases and risk factors for disease is very often obtained from self-reports of the target population. The data may be collected through face-to-face-interviews, telephone-interviews or mail-back questionnaires. The advantages of self-reports are substantially lower costs compared to clinical assessments, such that large, representative samples can be recruited. However, the accuracy of self-reports depends on respondents’ correct understanding of the medical condition, the ability to recall it, and the willingness to report it. Furthermore, diseases may cause symptoms without having been clinically confirmed. Hence, the validity of the information based on self-reports might be affected by random and systematic errors. The rate of incorrect reporting and therefore misclassification can vary by disease, patient characteristics or by the severity of the disease [1].

The prognosis and life-expectancy of breast cancer patients has improved due to advances in oncological therapy. The relative 5-year relative survival of patients with breast cancer continues to improve, and now exceeds 80% in European countries [2]. In addition to the age-related cardiovascular risks, late cardiac events induced by radiation therapy and chemotherapeutic treatment with anthracyclines must be considered in breast cancer survivors [3, 4]. The PASSOS-heart study investigated late cardiac events after breast cancer therapy. In this retrospective cohort study of women treated for localized breast cancer during 1998 and 2008 in Germany, the authors investigated whether contemporary 3D-conformal radiotherapy was associated with an elevated long-term cardiac morbidity risk [5]. A mail-back questionnaire assessed medical diagnoses of cardiac illness after cancer treatment and cardiovascular co-morbidities. After a median follow-up of 8.3 years, no evidence was found for a significantly elevated cardiac morbidity risk in women with radiotherapy for left-sided tumors who had, on average, much higher radiation exposure compared to women with radiotherapy for right-sided tumors: the hazard ratio (HR) for left-sided vs. right-sided tumors was 1.07 (95% confidence interval (CI) 0.89–1.29). The results were adjusted for age, chemotherapy and cardiovascular risk factors.

However, accurate information about the incidence of cardiac disease after breast cancer therapy is essential for the validity of the study results. The impact of misclassification should be evaluated and has to be considered in the critical interpretation of research results [6]. For that reason, we asked the general practitioners (GP) and other medical specialists for validation of patient-reported morbidity information. Since medical records themselves might be partially based on patient self-reports or may be incomplete, we did not treat either assessment method as the gold standard [7]. Rather, we examined their mutual agreement.

The following questions will be addressed.

1. How well do patient self-reports agree with their GPs’ information on the presence or absence of incident cardiac late effects after breast cancer therapy?
2. Which patient characteristics are associated with varying levels of agreement between self-reports and GP-information?
3. Does misclassification of cardiac events in patient self-reports affect the results of the morbidity analysis of the PASSOS-heart study?

## Methods

### Study population

**Fig. 1:**
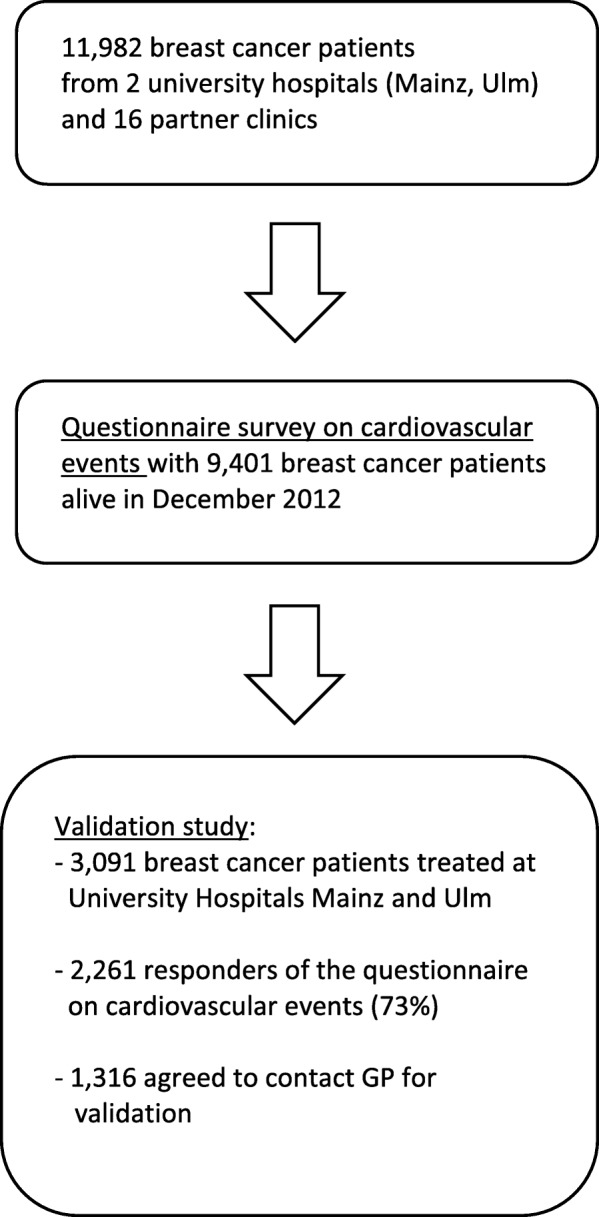
The PASSOS-Heart Study population and the validation sample

The PASSOS-Heart Study is a retrospective multicenter cohort study with a total of 11,982 women who met the inclusion criteria. Eligible breast cancer patients were diagnosed and treated between 1998 and 2008 at the Mainz University Medical Center, at the Ulm University Hospital, or at one of 16 smaller partner clinics. Inclusion criteria were a histologically confirmed primary and localized breast cancer disease, either an invasive carcinoma or a carcinoma in-situ. Women with primary metastatic disease or bilateral breast cancer were not included. An individual follow-up was carried out in order to assess the vital status on December 2012 [8]. At the end of the follow-up period, more than 78% of the women were still alive (*n* = 9401), 114 were lost to follow-up and 2467 were reported to be deceased. A subsample of 3091 women, who were treated at the two university clinics was considered for the validation study (Fig. 1).

### Questionnaire survey on cardiovascular diseases and risk factors

In 2014, a questionnaire was mailed to patients who were still alive (n = 9401). The questionnaire assessed which of the following events had ever been diagnosed by a physician: myocardial infarction (MI), angina pectoris, congestive heart failure, arrythmia, valvular heart disease, stroke. The questionnaire also asked whether any of the following diseases were ever diagnosed: diabetes mellitus, hypercholesterolemia (hyperlipoproteinemia), thyroid functional disease, chronic kidney disease, chronic obstructive pulmonary disease (COPD). For each cardiac event or cardiovascular co-morbidity, the women were asked the following question: “Did a physician ever diagnose one of the following diseases? If yes, when did you get the diagnosis for the first time? Please, indicate age or calendar year”. The questionnaire had to be sent back to the study center together with an informed consent (Additional file 1).

### Validation study: The general practitioner questionnaire

Patients who agreed to participate in the PASSOS-Heart Study were asked to provide contact information for their general practitioner or medical specialist (GP) and were asked for consent to have their GP contacted for validation of morbidity information. Due to budget constraints, only GPs of patients from Mainz University Medical Center and the Ulm University Hospital were contacted. Patients from the 16 partner clinics were not part of the validation study. The general practitioners received a written questionnaire about each patient who had given informed consent. Questions concerned the presence or absence of the same cardiovascular diseases that were included in the patient questionnaire, furthermore, the date of diagnosis and the appropriate ICD-code (International Classification of Diseases). In addition, we requested the time since the patient was under medical care in the doctor’s office.

### Statistical analysis

Cardiac events were only considered in the analysis if they reportedly had occurred after cancer therapy.

Descriptive statistics were used to describe the study population and morbidity data from both information sources. Responses were coded as a binary variable (yes: confirmation of having a particular diagnosis or no: lack of confirmation). A missing response was coded as lack of confirmation (no).

Rather than treating either assessment method as the gold standard, we examined the relative agreement between patients’ self-reports and GP information. This decision was based on the fact that patients permit contact either to a general practitioner, or to a medical specialist. Each medical doctor has a specific field of activity and possibly inconsistent or missing information on cardiovascular diseases and disease history. In our validation sample, a total of 60% of patients permitted contact to their GP or gynecologist, 32% to a cardiologist or internal specialist. However, chronic disease registration in the practice of a GP may not be complete. Furthermore, complaints or symptoms attributed to a specific disease by the patients may not be communicated to a GP [9].

For each endpoint, the agreement of the patients’ self-reports with the GP data was measured as the proportion of patients with identical information from both sources relative to all patients (correct classification rate, CCR). Positive agreement means that the disease of interest was noted as present by both, GP and patient. Negative agreement means that both data sources note a medical condition as absent. Over-reporting here means that a patient lists a medical diagnosis as present while the GP does not confirm this condition. Underreporting here means that a patient does not list a particular diagnosis as present while the GP does.

Cohen’s kappa coefficient was calculated for each individual endpoint and for a composite endpoint of any cardiac event to determine the chance-corrected agreement between self-reported questionnaire data and medical records. A kappa value of < 0.40 was considered poor-to-fair-agreement, 0.40 to < 0.60 considered moderate agreement, 0.60 to < 0.80 substantial agreement, 0.80 to 1.0 excellent agreement [10].

A multivariate Bayesian random effects logistic regression assessed the association of patient characteristics with the degree of agreement between self-reports and information from GPs over all individual diagnoses. Covariates included age at diagnosis of breast cancer (in years), calendar year of diagnosis of breast cancer, the duration of patient-physician contact, and CASMIN educational level [11]. The latter one was used as proxy for socioeconomic status of the study participants. Model fitting was implemented in the Stan Bayesian modeling language [12]. Model diagnostics assessed mixing of chains, autocorrelation, and convergence. 95% credible intervals were derived from the marginal posterior distributions of model coefficients.

The effect of misclassification of the outcome event on the risk estimate for left-sided vs. right-sided radiotherapy in Cox regression was analyzed in a simulation study with 1000 replications. Based on estimated probabilities of over-reporting and underreporting, the self-reported outcomes were randomly re-assigned in each replication. If re-assigning the outcome resulted in the presence of an event, a random survival time was simulated based on the individual predicted cumulative hazard function derived from the original Cox regression [5] If re-assigning the outcome resulted in the absence of an event, observation time was set to last until the date of censoring, i.e., the date of the questionnaire. In each simulation replication, the original Cox model was fit to the generated data from patients with radiotherapy. Aggregate coefficient estimates as well as their confidence interval bounds were then obtained by averaging over all replications.

Analyses were carried out in the statistical environment R [13].

## Results

### Validation sample and patients’ characteristics

A total of 3091 women were considered for the validation study, and 2261 (73%) women send back the questionnaires on cardiovascular events (Fig. 1). From these, 1316 patients (58.2%) gave consent to contact their respective GP. On average, women who gave consent to contact the GP were significantly younger at diagnosis (mean = 55.2 years) than those who denied contact to a physician (mean = 58.6 years, *p* < 0.0001). Patients who gave consent had chemotherapy less often, and radiotherapy more often compared to patients who did not consent (both p < 0.0001). Staging, type of surgery and history of a cardiac event before breast cancer diagnosis were equally distributed in both groups. **(**Table 1).

**Table 1:** Clinical characteristics of the study population stratified by permission to contact GP

Clinical characteristic		No Denied Consent	Yes Gave consent
Age at breast cancer diagnosis (years)	mean SD^a^	58.6 11.6	55.2 10.6
Age at questionnairesurvey (years)	Mean SD	69.4 11.7	65.9 10.7
Follow-up (years)	median	9.62	9.17
T-stage (N, %)	0/1 2 3 4 In situ Unknown	507 (53.7%) 264 (27.9%) 35 (3.7%) 30 (3.2%) 88 (9.3%) 21 (2.2%)	727 (55.2%) 380 (28.9%) 39 (3.0%) 25 (1.9%) 109 (8.3%) 36 (2.7%)
N-stage (N, %)	0 1 2 3 X	587 (62.1%) 217 (23.0%) 38 (4.0%) 27 (2.9%) 76 (8.0%)	829 (63.0%) 318 (24.2%) 58 (4.4%) 29 (2.2%) 82 (6.2%)
Chemotherapy (N, %)	Yes No Unknown	515 (54.5%) 399 (42.2%) 31 (3.3%)	604 (45.9%) 671 (51.0%) 41 (3.1%)
Radiotherapy (N,%)	Yes No Unknown	737 (78.0%) 166 (17.6%) 42 (4.4%)	1090 (82.8%) 174 (13.2%) 52 (4.0%)
Type of surgery (N, %)	None Breast conserving Mastectomy Unknown	3 (0.3%) 751 (79.5%) 190 (20.1%) 1 (0.1%)	8 (0.6%) 1067 (81.1%) 241 (18.3%) 0 (0.0%)
History of cardiac event (N, %)	Yes No	174 (18.4%) 771 (81.6%)	220 (16.7%) 1096 (83.3%)
Total *N* = 2261		945	1316

^a^*SD* Standard deviation

### Agreement for cardiac endpoints

From 1316 patients who agreed to have their GP contacted, medical records could be obtained from 703 practices for 1212 (92.1%) patients for validation of self-reported cardiac events after breast cancer therapy (myocardial infarction, angina pectoris, congestive heart failure, arrythmia, valvular heart disease). Table 2 presents the frequencies of agreement between patients’ and GPs’ information for the composite cardiac endpoint. The overall agreement between GP information and self-reports is 84.5% (1024 patients). The chance corrected agreement is poor to fair with a kappa of 0.35. Of 1055 breast cancer patients reporting no cardiac event, 950 (90%) had no such diagnosis in GPs’ medical record. There was 47% positive agreement regarding the occurrence of any cardiac endpoint: 157 breast cancer survivors indicated a cardiac event, and the same diagnosis was confirmed by GPs for 74 women.

**Table 2:** Agreement between patients’ questionnaire and GP information regarding composite endpoint of any cardiac event

	GP^a^: negative n (%)	GP: positive n (%)	All N (%)
Patients questionnaire: negative	950 (90%)	105 (10%)	1055 (100%)
Patients questionnaire: positive	83 (53%)	74 (47%)	157 (100%

Correct Classification Rate: 84.5%, kappa 0.35

^a^*GP* general practitioners’ information

Table 3 presents the agreement between patients’ questionnaire and GP information regarding specific cardiovascular endpoints. The presented outcomes include stroke even though stroke was not a cardiac event, but has been considered as a secondary outcome in the PASSOS-heart study.

**Table 3:** Proportions of agreement and disagreement between patients questionnaire and general practitioners information regarding incident cardiovascular endpoints

Disease	GP^a^, negative P^b^ negative (Agreement)	GP, positive P positive (Agreement)	GP positive P negative (Underreporters)	GP negative P positive (Overreporters)	Kappa	CCR^c^
Stroke	968 (96.9%)	14 (1.40%)	7 (0.70%)	10 (1.00%)	0.61	98.3%
Myocardial infarction	1003 (99.2%)	3 (0.30%)	1 (0.10%)	4 (0.40%)	0.54	99.5%
Angina pectoris	935 (95.0%)	14 (1.42%)	28 (2.83%)	11 (1.11%)	0.40	96.1%
Valvular heart disease	913 (93.7%)	16 (1.64%)	34 (3.50%)	11 (1.13%)	0.39	95.4%
Arrythmia	836 (88.8%)	27 (2.87%)	41 (4.36%)	37 (3.93%)	0.37	91.7%
Congestive heart failure	905 (93.5%)	14 (1.45%)	21 (2.17%)	28 (2.89%)	0.34	94.9%

^a^*P* patients’ questionnaire

^b^*GP* general practitioners’ information

^c^*CCR* correct classification rate

The highest proportion of positive agreement is observed for arrhythmia. From a total of 941 patients, 2.87% reported an existing diagnosis that was confirmed by the GP while negative agreement was 88.8%. However, for myocardial infarction (MI) only 0.30% reported an existing diagnosis that was confirmed by the GP while negative agreement was 99.2%. The proportion of under-reporters ranges from 0.10% for MI to 4.36% for arrhythmia. Proportions of over-reporters are similar, ranging from 0.40% for MI to 3.93% for arrhythmia.

Overall, moderate to substantial agreement between self-reports and medical records is found for MI (kappa 0.54) and furthermore for stroke (kappa 0.61). Poor to fair accuracy is present for angina pectoris, valvular heart disease, arrhythmia and congestive heart failure.

### Determinants for the agreement regarding any cardiac event

For the combined endpoint of any cardiac event, agreement between patient self-reports and information from GPs was negatively associated with higher age (Odds Ratio (OR) 0.95, 95% CI 0.93–0.97), and positively associated with a more recent year of cancer diagnosis (OR 1.1, 95% CI 1.01–1.13) as well as with higher socioeconomic status respectively educational level (OR 1.2, 95% CI 0.73–1.90). Agreement was higher when patients were treated by their GPs since the initial date of the patients’ breast cancer diagnosis (OR 1.4, 95% CI 1.01–2.07).

### Agreement for cardiovascular risk factors

The morbidity analysis of the PASSSOS-heart study adjusted for confounders like several potential cardiovascular co-morbidities. A moderate agreement between information from patients and GP was observed for hypercholesteremia (kappa 0.40) and chronic kidney disease (kappa 0.40). Substantial agreement could be determined for diabetes (kappa 0.78), hypertension (kappa 0.71) and thyroid functional disease (kappa 0.62) (Table 4).

**Table 4:** Proportions of concordance and discordance between patients questionnaire and GP regarding specific prevalent cardiovascular co-morbidities

Disease	GP^a^, negative P negative (Agreement)	GP, positive P positive (Agreement)	GP negative P positive (Overreporters)	GP positive P negative (Underreporters)	Kappa	CCR^b^
Diabetes mellitus	866 (86.3%)	93 (9.26%)	7 (0.70%)	38 (3.79%)	0.78	95.5%
Hypertension	492 (49.0%)	370 (36.9%)	53 (5.28%)	89 (8.86%)	0.71	85.9%
Thyroid functional disease	508 (50.5%)	315 (31.3%)	134 (13.3%)	49 (4.87%)	0.62	81.7%
COPD	839 (84.0%)	72 (7.21%)	51 (5.11%)	36 (3.61%)	0.57	91.3%
Hyper-Cholesteremia	395 (40.3%	294 (30.0%)	99 (10.1%)	193 (19.7%)	0.40	70.2%
Chronic kidney disease	905 (93.1%)	18 (1.90%)	18 (1.90%)	31 (3.20%)	0.40	95.0%

^a^*GP* general practitioners’ information

^b^*CCR* correct classification rate

### Effects of event misclassification on cox regression coefficients

Results from the simulation study suggest that with the given data, taking into account event misclassification does not change the evaluation of tumor laterality as a non-significant risk factor for the incidence of late cardiac events in the PASSOS-heart study [5]. The average HR for left-sided radiotherapy over all simulation replications was 1.03 (95% CI 0.87–1.22).

## Discussion

### Main findings

In a validation study with breast cancer survivors, we assessed the agreement between patients’ questionnaire responses on cardiac events after therapy and corresponding information obtained from GPs. For the composite endpoint of any cardiac disease, a total of 1055 breast cancer patients reported no cardiac event. For 950 patients (90%) this information was confirmed by GPs’ medical record. There was 47% positive agreement for the occurrence of a cardiac event: 157 breast cancer survivors indicated a cardiac event, and the same diagnosis was confirmed by GPs for 74 women. For specific diagnoses, the study showed moderate to substantial agreement for MI but only poor to fair agreement for angina pectoris, valvular heart disease, arrhythmia and congestive heart failure. Factors associated with higher agreement were (younger) age, a more recent cancer diagnosis, higher socioeconomic status, and longer patient-physician contact. In addition, cardiovascular risk factors and co-morbidities have been assessed. The agreement was highest for diabetes and hypertension. This validation analysis was based on data from the PASSOS-heart study. A re-analysis confirmed the main findings of the PASSOS-heart study: even when taking into account the possibility of endpoint misclassification, no evidence for a significantly elevated cardiac morbidity risk in patients with left-sided vs. right-sided radiotherapy could be found.

### Comparison with other validation studies

Our results show that earlier reports can be confirmed in a different health care system. In a validation study with 357 Danish breast cancer patients who conducted a telephone-interview on cardiovascular diseases (CVD) [14], the authors found a high accuracy (94%) for reporting the absence of CVD, comparing self-reports and diagnostic codes from the Danish National Patient Register. This is in line with our and previous studies, who showed a high accuracy of recall for participants who did not report a CVD [15–17]. Furthermore, it was found that when specific self-reported diagnoses (such as angina pectoris) could not be found in the registry, other CVDs were recorded. Complex diagnostic criteria and closely related cardiac symptoms could make it difficult for patients to report specific diagnoses accurately. Combining closely related cardiac diseases (angina pectoris and myocardial infarction) may increase the validity of self-reports [14, 17]. Accordingly, in our validation study single cardiac diseases were combined to a comprehensive item. A large study with 1936 US patients from the California Breast Cancer Survivorship Consortium (CBCSC) compared self-reports and electronic medical records for cardiovascular comorbidities [6]. For MI a kappa value of 0.66 was found. This is in line with the results of our study (kappa value for MI: 0.54). MI is a severe event requiring medical care or hospitalization. However, it should be noted that breast cancer patients might a have a greater awareness of symptoms for possible late effects related to cancer therapy and may be more health conscious in general compared to non-cancer patients. Thus, variability in published study results might be due to different study populations. However, a population-based study found a kappa value of 0.80 for a history of MI and of 0.71 for stroke (questionnaire vs. medical record) [15]. Hence, MI and stroke could be recalled reliably. Both conditions are associated with pronounced symptoms and medical after care and may be more likely to be reported correctly [18]. Poor agreement between self-report and medical documentations for other heart diseases (arrhythmia, congestive heart failure, angina pectoris) might be due to lack of awareness or underreporting due to non-specific symptoms [19]. Self-reports on medical conditions that are well defined and easily diagnosed have a good agreement, in contrast to conditions characterized by complex, non-specific symptoms [15].

Hypertension, hypercholesterolemia and diabetes are important chronic diseases and risk factors for cardiac events. Screening for such cardiovascular risk factors could be obtained by self-reports of the target population. Regardless of different methodologies and study populations, there are common findings across studies. For diabetes and hypertension, a good agreement was demonstrated in many validation studies. Health conditions requiring regular self-monitoring, frequent interaction with the helthcare system or medication might increase awareness and consequently improve recall of the disease [2, 7, 13–15]. Accuracy for self-reported hypercholesterolemia was find to be lower (kappa value 0.37) than that for hypertension (kappa 0.61) and diabetes (kappa 0.76) [20]. The results of our validation study correspond approximately to these findings.

To assess their accuracy, self-reports can be compared with medical records or information on specific diagnoses provided by physicians. Limitations of medical records may be inconsistent documentation standards or underreporting of pre-admission conditions. On the other hand, diagnoses provided by physicians might be incomplete due to missing information concerning earlier diseases diagnosed by a previous physician [7]. Furthermore, the precise wording in which the co-morbidity questions were asked might be an important factor for agreement or non-agreement between self-report questionnaire and GPs information. The presented results might differ from other validation studies, which have used a different manner of data collection in their questionnaires. Finally, several factors might influence the validity of self-reported medical conditions. In our study, we found that younger age and higher education were found to be associated with greater total agreement. However, an influence of age and/or education was detected in some [7, 19, 20], but not all studies [6].

In summary, the accuracy of morbidity data from patient self-reports is determined by multiple factors including patients’ characteristics, reporting methods, type of diagnosis and presence of distinct disease criteria. Furthermore, different comparison tools could impede the comparability of published results [14].

### Strengths and limitations

The present analysis of self-reported cardiac events and cardiovascular risk factors is based on a large validation sample of 3091 women. A representative sample of 73% (*n* = 2.261) responded to a detailed health questionnaire-survey. Available data allowed for the analysis of discrepancies between questionnaire data and GP information, including age, time since cancer diagnosis, socioeconomic status, and details of breast cancer therapy.

However, it should be noted that the validation study itself is a reduction in sample size, which might be associated with a selection of patients. The PASSOS-Heart study comprise 9.401 women (Fig. 1), being still alive at the end of the observational period. Those who did not respond to the questionnaire had less favorable staging with significantly less frequent T0/T1 or N0 status compared to the responding women. Furthermore, adjusting for age at diagnosis, non-responders had a significantly lower proportion of pre-existing cardiac comorbidity at the time of diagnosis [5]. Furthermore, the women in our validation sample are further selected for having given consent to request medical records from their GP. From 2.261 women who participated in the questionnaire-survey, only 1316 (58.2%) gave consent to contact their respective GP. Patients who gave consent had chemotherapy less often compared to patients who did not consent. Cardiotoxic chemotherapy agents like anthracyclines have been identified as a potential cause of cardiovascular disease [4]. Furthermore, women who gave consent to contact the GP were younger at breast cancer diagnosis than those who denied contact to a physician. The incidence of cardiac disease increase with increasing age. Thus, regarding the study sample considered for the validation study, a selection bias of women who are healthier cannot be excluded.

Furthermore, generalization of the study results might be limited due the restriction to female breast cancer survivors. It has been shown in a sample of population residents that women had highger agreement for heart failure and MI when comparing self-report questionnaires with medical records data [15]. On the other hand, in a hospital-based study with patients diagnosed with acute coronary syndrome, the agreement varied not significantly by sex [19].

Another limitation of this validation study might be that a missing response of a patient or GP regarding a certain disease was coded the same as a confirmed absence of a disease. The rationale for this coding strategy is that a missing response may result from both - incomplete information or from an active diagnostic process where no signs of a disease were detected. Both possibilities are indistinguishable in our data. Among the study participants, we observed a total of 41 missing information on cardiac events, in contrast to the GP, who confirmed a cardiac disease. Vice versa, for 29 missing values from GPs, the breast patients specified a diagnosis on cardiac disease. Due to the small numbers (*n* = 70/1.212), we did not suppose a distortion of our results due to misclassification.

## Conclusions

Questionnaire-based, self-reported medical diagnoses among female breast cancer survivors can provide sufficiently valid data especially for conditions with pronounced symptoms like stroke or myocardial infarction. The agreement for cardiovascular risk factors was highest for diabetes and hypertension. However, information bias should be considered for health conditions that might require less monitoring, or are less defined by acute events. Complex cardiac symptoms (e.g. angina pectoris, congestive heart failure, ischemic heart disease) are difficult to classify reliably in observational studies. In order to reduce misclassification, it could be recommended for questionnaire-based investigations to assess whether patients ever had any cardiac or cardiovascular condition. In summary, self-reporting of health conditions is a useful assessment method to detect late cardiac effects in breast cancer patients. A re-analysis of the PASSOS-heart study on cardiac late events among breast cancer patients confirmed the main results, and were not affected by possible misclassification.

## Additional file

Additional file 1: Patient-Questionnaire of the PASSOS-Heart Study. (DOCX 14 kb)

## Abbreviations

CCR: Correct Classification Rate
CI: Confidence Limits
GP: General Practitioner
HR: Hazard Ratio
MI: Myocardial Infarction
OR: Odds Ratio
PASSOS-Heart study: Personalized assessment of late health risks after radiation exposure for optimization of medical applications in medicine
