# Supplementary material for: Cardiac late events in German breast cancer patients: a validation study on the agreement between patient self-reports and information from physicians
Source: BMC Cardiovasc Disord. 2018 Nov 29;18:218. doi: 10.1186/s12872-018-0961-7 (PMC6267788; doi:10.1186/s12872-018-0961-7)
Supplement: Supplementary file 1 — Patient-Questionnaire of the PASSOS-Heart Study. (DOCX 14 kb) [file 12872_2018_961_MOESM1_ESM.docx]

**Supplement to manuscript BCAR-D-18-00198:**

Patient-Questionnaire of the PASSOS-Heart Study

| **Did a physician ever diagnosed one of the following diseases?**  (multiple answers possible**)** |  |  | **If yes, when did you get the diagnosis for the first time?**  (Please, indicate age **or** calendar year) | |
| --- | --- | --- | --- | --- |
|  | No | Yes | Age | Calendar Year |
| Myocardial infarction | □ | □ | \|__\|\|__\|  Year | \|__\|__\|__\|__\|  (Year) |
| Angina pectoris | □ | □ | \|__\|\|__\|  Year | \|__\|__\|__\|__\|  (Year) |
| Congestive heart failure  (ischemic heart disease) | □ | □ | \|__\|\|__\|  Year | \|__\|__\|__\|__\|  (Year) |
| Arrythmia of the heart | □ | □ | \|__\|\|__\|  Year | \|__\|__\|__\|__\|  (Year) |
| Valvular heart disease | □ | □ | \|__\|\|__\|  Year | \|__\|__\|__\|__\|  (Year) |
| Stroke | □ | □ | \|__\|\|__\|  Year | \|__\|__\|__\|__\|  (Year) |
| Diabetes mellitus | □ | □ | \|__\|\|__\|  Year | \|__\|__\|__\|__\|  (Year) |
| Hypertension | □ | □ | \|__\|\|__\|  Year | \|__\|__\|__\|__\|  (Year) |
| Increased blood cholesterol,  increased blood lipids | □ | □ | \|__\|\|__\|  Year | \|__\|__\|__\|__\|  (Year) |
| Chronic Lung Disease  (Asthma, Bronchitis, Chronic obstructive pulmonary disease | □ | □ | \|__\|\|__\|  Year | \|__\|__\|__\|__\|  (Year) |
| Chronic kidney disease | □ | □ | \|__\|\|__\|  Year | \|__\|__\|__\|__\|  (Year) |
| Thyroid functional disease | □ | □ | \|__\|\|__\|  Year | \|__\|__\|__\|__\|  (Year) |

Did you have a pacemaker? No, don’t know, yes, if yes-when (calendar year)?

Did you have a stent implantation? No, don’t know, yes, if yes-when (calendar year)?

Was a balloon dilatation done? No, don’t know, yes, if yes-when (calendar year)?

Was a pass surgery done? No, don’t know, yes, if yes-when (calendar year)?
